# TROPOMI enables high resolution SO_2_ flux observations from Mt. Etna, Italy, and beyond

**DOI:** 10.1038/s41598-018-37807-w

**Published:** 2019-01-30

**Authors:** Manuel Queißer, Mike Burton, Nicolas Theys, Federica Pardini, Giuseppe Salerno, Tommaso Caltabiano, Matthew Varnam, Benjamin Esse, Ryunosuke Kazahaya

**Affiliations:** 1 School of Earth and Environmental Sciences, University of Manchester, Oxford Road, Manchester, M139PL UK; 2 Royal Belgian Institute for Space Aeronomy (BIRA-IASB), Ringlaan-3-Avenue Circulaire B-1180 Brussels, Brussels, Belgium; 3 Istituto Nazionale di Geofisica e Vulcanologia, Sezione di Catania, Piazza Roma, 2, 95123 Catania, Italy; 4 Geological Survey of Japan, AIST, 1-1-1 Higashi, Tsukuba, Ibaraki, 305-8567 Japan

## Abstract

The newly launched imaging spectrometer TROPOMI onboard the Sentinel-5 Precursor satellite provides atmospheric column measurements of sulfur dioxide (SO_2_) and other gases with a pixel resolution of 3.5 × 7 km^2^. This permits mapping emission plumes from a vast number of natural and anthropogenic emitters with unprecedented sensitivity, revealing sources which were previously undetectable from space. Novel analysis using back-trajectory modelling of satellite-based SO_2_ columns allows calculation of SO_2_ flux time series, which would be of great utility and scientific interest if applied globally. Volcanic SO_2_ emission time series reflect magma dynamics and are used for risk assessment and calculation of the global volcanic CO_2_ gas flux. TROPOMI data make this flux time series reconstruction approach possible with unprecedented spatiotemporal resolution, but these new data must be tested and validated against ground-based observations. Mt. Etna (Italy) emits SO_2_ with fluxes ranging typically between 500 and 5000 t/day, measured automatically by the largest network of scanning UV spectrometers in the world, providing the ideal test-bed for this validation. A comparison of three SO_2_ flux datasets, TROPOMI (one month), ground-network (one month), and ground-traverse (two days) shows acceptable to excellent agreement for most days. The result demonstrates that reliable, nearly real-time, high temporal resolution SO_2_ flux time series from TROPOMI measurements are possible for Etna and, by extension, other volcanic and anthropogenic sources globally. This suggests that global automated real-time measurements of large numbers of degassing volcanoes world-wide are now possible, revolutionizing the quantity and quality of magmatic degassing data available and insights into volcanic processes to the volcanological community.

## Introduction

After water vapor and CO_2_, SO_2_ is usually the most abundant volcanic gas, exsolving during depressurization of magma^1,2^. Measured SO_2_ mass fluxes reflect magma dynamics, decompression and crystallization processes^2–8^. For example, SO_2_ fluxes contain information about magma-gas separation depths, conduit structure and permeability and magma pressure^2,5^. Moreover, eruption risk, eruption style and strength can be inferred using SO_2_ fluxes^2,5,8^, providing a crucial resource for volcanic risk managers. SO_2_ flux is also used together with gas ratio data (e.g. CO_2_/SO_2_ mass ratios) to determine the fluxes of other volcanic gases, which provides the underpinning for our understanding of global volcanic volatile budgets^9^. As there is no significant natural SO_2_ background and owing to distinct absorption features in the ultraviolet (UV) and infrared wavelength ranges, subaerial volcanic SO_2_ is relatively easy to detect. SO_2_ is thus a precious and widely used proxy of volcanic activity and routinely measured with ground-based optical instruments^2,10–13^. While ground-based SO_2_ measurements have allowed priceless insights into the inner workings of volcanoes and the effect of volcanic SO_2_ degassing on the Earth system, most volcanoes still lack an observational database^14,15^, particularly in regions that have highly active but remote volcanic emitters such as Indonesia or the Kuril Islands and Kamchatka^16,17^.

Satellite remote sensing may help overcoming observational biases by continuous monitoring on a global scale, extending monitoring capabilities to any SO_2_ emitter in the world^18–20^. There exists a variety of satellite sensors that can detect volcanic SO_2_ atmospheric abundance and which have been used over the past decades, including the Total Ozone Mapping Spectrometer^21^ (TOMS), the Global Ozone Monitoring Experiment-2^22^ (GOME-2), the Hyperspectral Infrared Atmospheric Sounder^23^ (IASI), the Ozone monitoring instrument^24^ (OMI) and recently the Tropospheric Monitoring Instrument TROPOMI^25,26^. The TROPOMI imaging spectrometer onboard the Sentinel-5 Precursor satellite represents a step-change in gas monitoring from space as it measures in four different spectral regions (UV, visible, near-infrared, shortwave infrared) and other than SO_2_ detects nitrogen dioxide, ozone, formaldehyde, methane and carbon monoxide (CO). Launched on a polar orbit, TROPOMI has a repetition time of 1 day and a swath width of 2600 km. Using a push-broom scanning technique the instrument provides images with a minimum pixel size of 7 × 3.5 km^2^, smaller than the minimum pixel size of 13 × 24 km^2^ of the predecessor OMI^27^, yet at a comparable spectral resolution and detection limit. IASI has a slightly larger footprint than TROPOMI (12 km diameter), but a relatively low sensitivity to surface SO_2_ enhancements, due to low thermal contrast and high light absorption by atmospheric water vapor in the infrared^28^. This makes TROPOMI the satellite SO_2_ sensor with currently the highest per pixel sensitivity to SO_2_. Volcanic SO_2_ concentrations previously undetectable by satellite may therefore now be measurable^26^. Another advantage of the increased spatial resolution of TROPOMI is that the filling of the measured pixels with a volcanic plume is higher than is the case for lower spatial resolution instruments. This is a major benefit as the SO_2_ signal is effectively less diluted with clean air and higher concentrations are observed. This allows TROPOMI to map volcanic plumes from space in unprecedented detail, separating volcanic plumes from nearby emitters, including other volcanic plumes, and providing details within a single plume. Plumes from passively degassing volcanoes can now be measured, and the snapshot of the plume acts as a recorder of the SO_2_ fluxes over the previous hours.

Knowledge of the plume height is key in the quantification of SO_2_ abundances in TROPOMI data, as the instrumental SO_2_ response is height dependent. Our aim is to produce robust SO_2_ flux time series using plume height corrected SO_2_ data from TROPOMI for volcanoes globally. A vital step towards this aim, and motivation of this study, is to validate the TROPOMI SO_2_ flux time series through comparison with high quality ground-based flux data.

**Figure 1:**
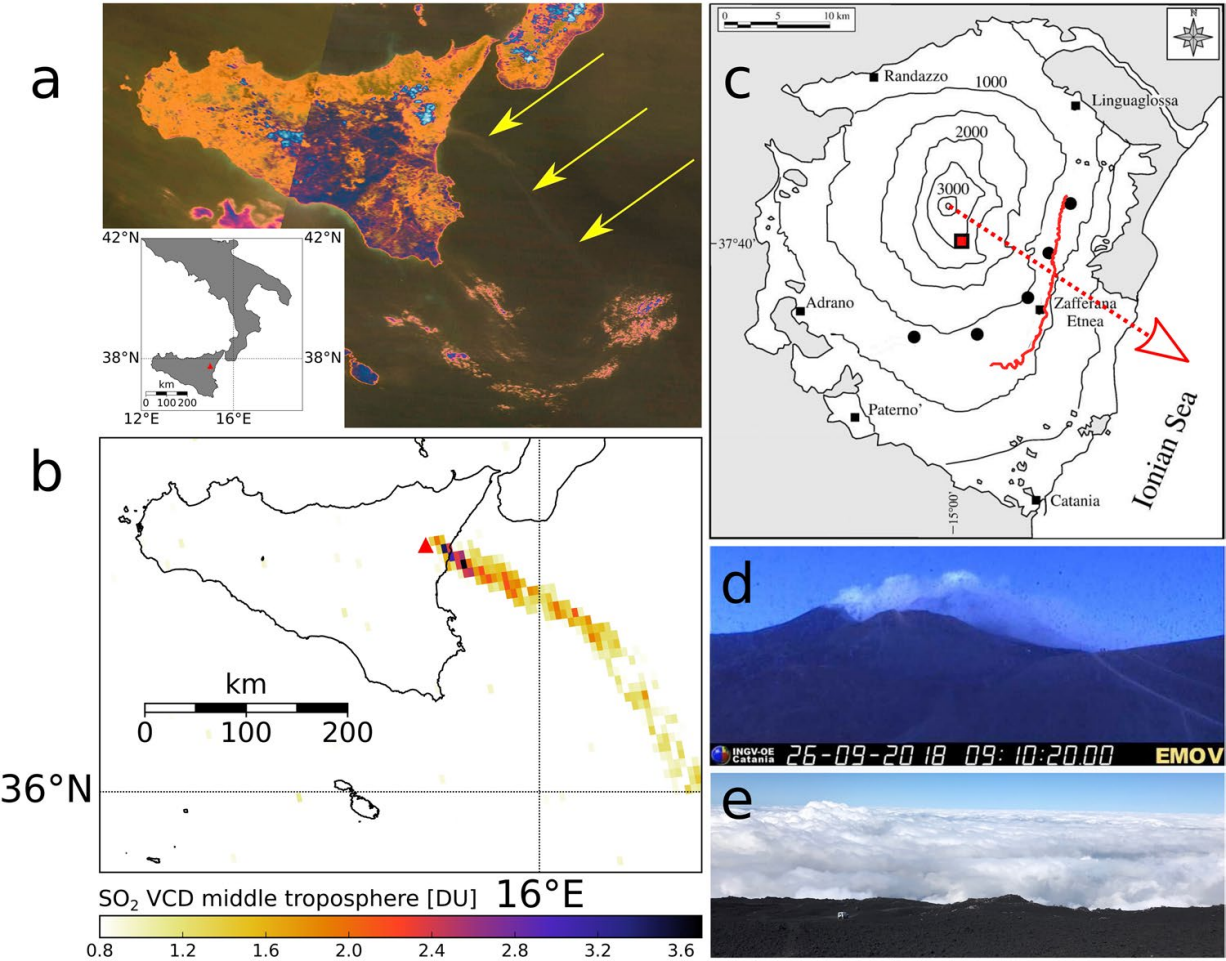
Probing Mt. Etna from space and ground. (**a**) Inset: Geographic setting of Sicily and Mt. Etna (red triangle) and MODIS Terra true color satellite image from 27 July 2018 showing a clearly visible plume emerging from Mt. Etna (marked by arrows). The color has been adjusted for better visibility of the plume. (**b**) TROPOMI data acquired on 27 July 2018 12h13 UTC with the native grid resolution, showing column densities assuming plume heights in mid-troposphere (6.5 to 7.5 km altitude) in Dobson units (DU). A threshold of 0.8 DU has been applied for display purposes. Cloud cover (cloud fraction 0.5) around the vent likely caused near-vent pixels with concentrations below 0.8 DU. (**c**) Location of FLAME scanners (black-solid circles). Black-solid squares indicate the main surrounding villages. The network was installed on the southern and eastern flanks of the volcano according to the western prevailing trade-wind. The red line marks the track of the ground traverse on 26 September 2018. The red arrow indicates the prevailing plume azimuth during the traverse (between 108° and 138°). The red square marks the location of the visible camera at the at the INGV Montagnola observatory (EMOV). (**d**) Image from the visual camera INGV network on 26 July 2018 taken from Montagnola (~2600 m asl) showing a condensing plume emerging from summit craters. (**e**) Photo taken from the summit of Mt. Etna on 26 July 2018 looking down on the clouds.

Mt. Etna, a multi-crater stratovolcano complex located at the east coast of Sicily in southern Italy (Fig. 1a) is one of the strongest volcanic sources of SO_2_ in the world^19^ producing voluminous degassing both between and during eruptions. Like most volcanic sources, Etna exhibits a high variability in quiescent emission strength^29^. The automated scanning FLAME (FLux Automatic MEasurements) network of currently nine UV spectrometers installed on the flanks of Mt. Etna delivers continuous SO_2_ concentration and flux measurements since 2006^29^, making Mt. Etna an ideal laboratory to compare volcanic SO_2_ fluxes derived from satellite data with ground-based measurements^30,31^.

Remote sensing instruments, including TROPOMI and FLAME, do not directly measure gas fluxes but Vertical Column Densities (VCDs, Fig. 1b) that need to be converted to fluxes using another data processing step, which adds further uncertainty to the final result. Comparing fluxes from TROPOMI and FLAME therefore combines the performance of both the instrument and post-processing. For TROPOMI data this post-processing is performed with the trajectory-modeling scheme PlumeTraj, which was previously used to retrieve plume heights and reconstruct SO_2_ flux time series from satellite data of eruptive volcanic SO_2_ degassing^32,33^. For FLAME, measured spectra are converted to SO_2_ column densities using an artificial background spectrum approach^29^. Fluxes are determined using an empirical relationship between plume velocity, derived from wind field forecasts, and plume height (Methods and Materials). This approach has several error sources, and we therefore also performed the most robust SO_2_ flux measurements possible, performing intensive ground-based traverse measurements with a USB2000 + spectrometer whilst measuring the plume velocity at the vent with video camera footage (Methods and Materials).

TROPOMI was launched in 2017 and has been through a commissioning phase, with data becoming available in October 2018. Consequently, only a few months of data are available and data exploitation is still in an exploratory stage. Here, we compare 34 days (5 July to 7 August 2018) of SO_2_ fluxes retrieved from TROPOMI data with ground-based measurements from the FLAME network (Fig. 1c) and fluxes from driving traverse measurements underneath the volcanic plume from 25 and 26 September 2018 (Fig. 1c).

Due to the mainly quiescent volcanic degassing level, fluxes as low as 160 t/day have been detected by FLAME and successfully retrieved from the TROPOMI data. A good match is found for most of the time series between all three datasets, which suggests that TROPOMI data combined with trajectory modeling represents a viable method to produce flux time series from TROPOMI data in quasi real-time, even for weakly degassing sources. We highlight that averaging of SO_2_ imagery can yield higher sensitivities^19^, but only at the expense of temporal variability information, which we believe is of high scientific value and critical for volcano surveillance. The capacity of TROPOMI to deliver intra-day flux time series from individual images is therefore remarkable and opens a new frontier in volcano monitoring and volcanic research.

Mt. Etna has multiple degassing vents in its summit craters. Between 5 July and 7 August, Mt. Etna experienced an intense period of eruptive activity from its summit craters, which spanned from explosive to lava effusion. Most of the activity occurred at Bocca Nuova and North East Craters (BN and NEC, respectively), which consisted of a continuous stage of low-energy explosive Strombolian activity confined within the craters. Between 23 and 29 August, after several months of quiet conditions, eruptive activity resumed also at South East Craters (SEC), with sporadic ash emission coupled with lava effusion. However, the SO_2_ degassing level remained at mean-high values with respect to the typical quiescent degassing level.

## Results

### SO_2_ fluxes from TROPOMI versus FLAME

**Figure 2:**
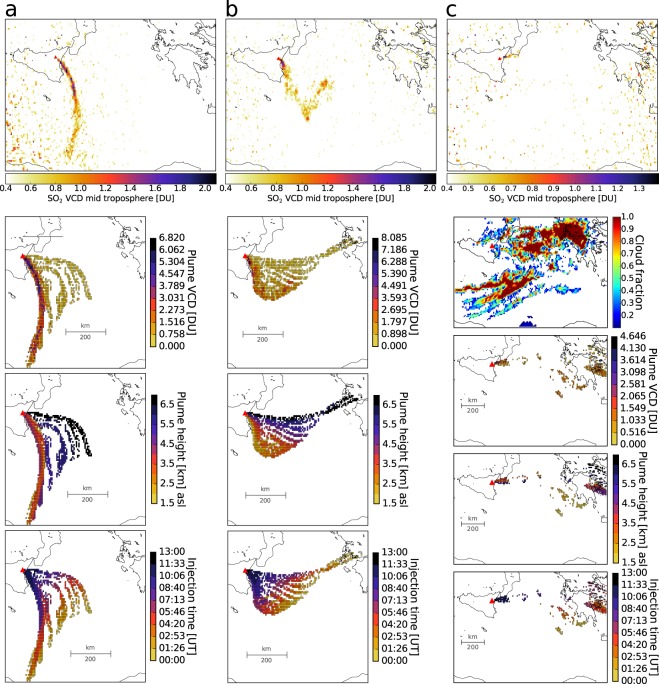
Examples of TROPOMI input satellite data used by PlumeTraj regridded (0.07° by 0.07°, approx. 7 × 7 km^2^) and with 0.4 DU threshold (twice the TROPOMI detection limit^25^) and PlumeTraj results: corrected VCDs (plume VCDs), plume height and injection time for filtered pixels (plume pixels). (**a**) 13 July 2018. (**b**) 12 July 2018. (**c**) 23 July 2018 with cloud fractions.

**Figure 3:**
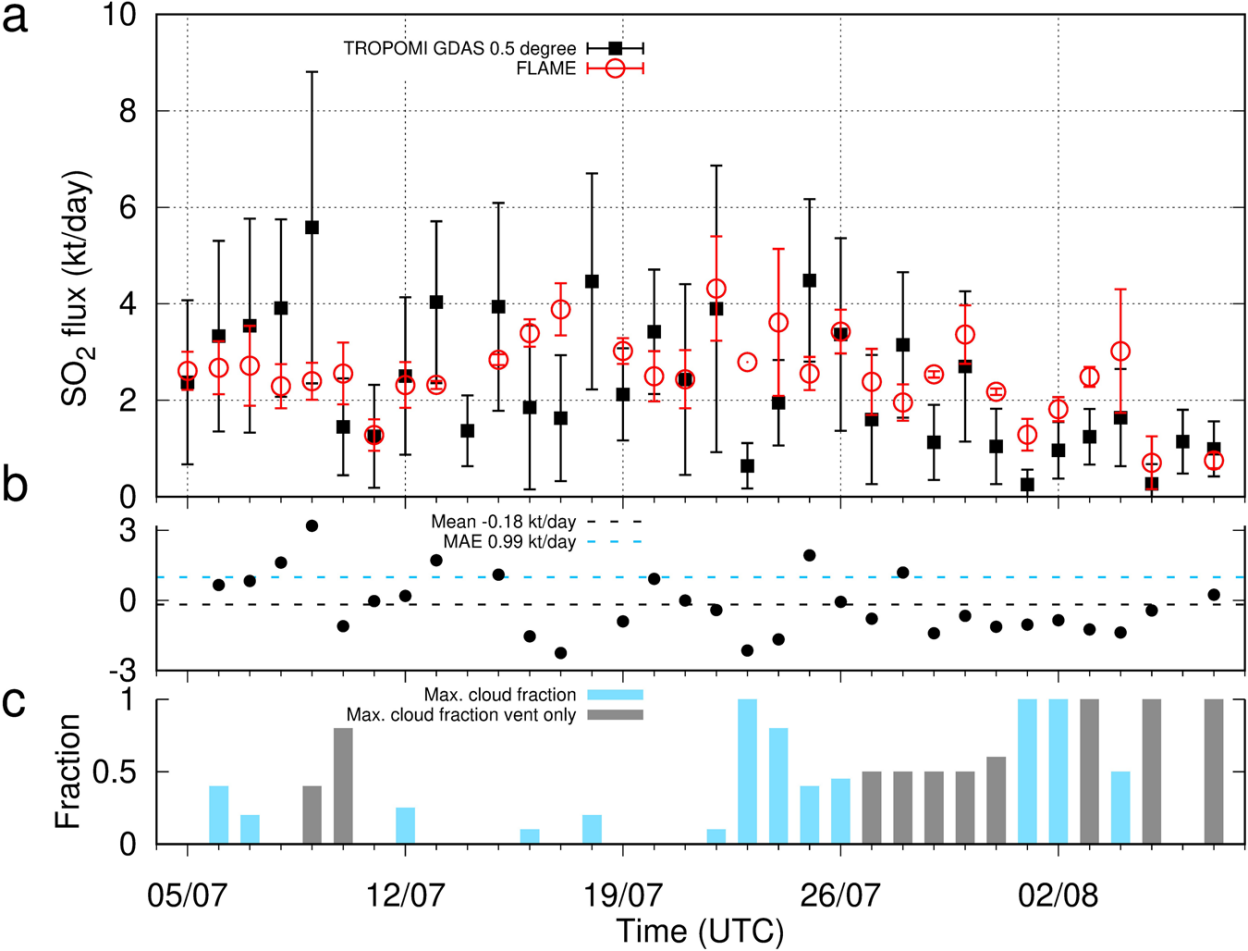
Time series of SO_2_ fluxes between 5 July and 7 August 2018. No FLAME data were recorded during 14, 18 and 23 July and 6 August. (**a**) Daily means of SO_2_ fluxes. PlumeTraj used TROPOMI data from midnight until noon, the time of the satellite overpass near the location of Mt. Etna, while FLAME only provides data after sunrise. Therefore, only those TROPOMI derived fluxes after 5 AM UTC and FLAME derived fluxes before 12h30 are used to compute the means (except for 13 and 23 July due to lack of FLAME data). Only one FLAME flux value was available for 23 July. (**b**) Residuals (TROPOMI – FLAME). MAE: mean average error. (**c**) Maximum cloud fractions co-located with plume. Days with cloud cover near the vent (gray boxes) were clear away from the vent.

**Figure 4:**
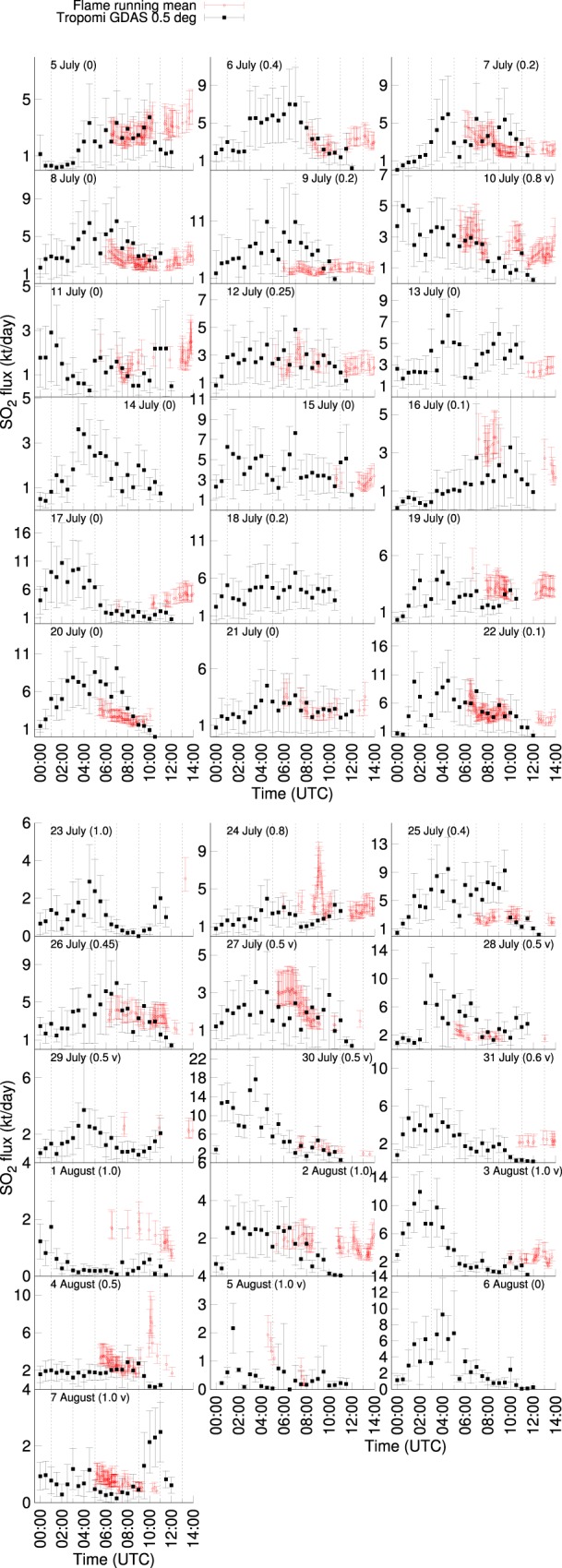
Intra-day comparison of data shown in Fig. 3. The numbers in brackets indicate maximum cloud fraction as in Fig. 3, v- only vent area affected). The earliest FLAME recordings began at around 6 AM. For some days (14, 18 and 23 July and 6 August) or parts of the day no FLAME data were available. A running mean over five time steps has been applied to the ~6 minute time resolution FLAME data to make it comparable to the 30 min resolution of the TROPOMI derived fluxes.

In the Mediterranean, clear sky conditions are common during the summer. To minimize cloud interference we therefore focus on the period between 5 July 2018 and 7 August 2018. Figure 2 shows TROPOMI data as used by PlumeTraj and the results: plume height corrected VCDs, corresponding plume heights and injection times, which were used to compute SO_2_ flux time series (Figs 3 and 4) as detailed in Material and Methods. Westerly winds dominated and caused southeastward dispersing, elongated plumes (Fig. 2a,b), until 4–7 August when major portions of the plume dispersed towards North West and North of Mt. Etna, an area not covered by the FLAME scanners (Fig. 1c). This explains the absence of FLAME data for days after 4 August (Fig. 3a).

The SO_2_ flux averaged over all data of the full month for TROPOMI data is 2.83 ± 1.66 kt/day and agrees with the average for FLAME (2.39 ± 1.09 kt/day). The daily averages of SO_2_ fluxes from FLAME and TROPOMI agree within the uncertainty for 25 out of 31 days (81%), but strong intra-day variability and errors mean the degree of linear correlation is relatively low (Pearson’s correlation coefficient 0.31). It is particularly encouraging that the trending between the data is compatible (Fig. 3a), such as the periodicity of ~7 days seen in both datasets, and the slight decrease in SO_2_ flux after 26 July. Also evident is an unusually low SO_2_ degassing strength between 5 and 7 August that both approaches picked up (Fig. 3a). The mean difference between fluxes derived from TROPOMI and FLAME is −0.18 kt/day (Fig. 3b). The absolute flux difference is 0.99 kt/day on average (mean absolute error, MAE, Fig. 3b).

Although for some days the plume shape indicated a relatively complex wind field, such as 12 July (Fig. 2b), the agreement with FLAME is fair. This is an important result as this indicates that computational less expensive, quasi real time flux retrievals may be achieved with reasonable confidence using the back-trajectory analysis performed by PlumeTraj.

On a 30 min time scale (Fig. 4), SO_2_ fluxes from TROPOMI data are compatible with the FLAME time series for 20 days (or 65%). Although confirmed by the FLAME result multiple times (6, 7, 8, 26, 28 July), the occasional symmetric, bell-like shape of the flux time series (e.g. 6 July, Fig. 4) may partly be caused by geometrical spreading of the plume and the associated dispersion of SO_2_, diluting SO_2_ concentrations per pixel below the detection limit of TROPOMI as a function of dispersion time.

Upon converting TROPOMI VCDs to fluxes, PlumeTraj contributes to the total uncertainty of the fluxes due to erroneous back-propagation of pixels to the vent due to wind-field data errors, introducing greater uncertainty in plume height and injection time. Depending on the day and time considered, the corresponding share was between 1% and 97% of the total error, the rest being contributed by uncertainties in TROPOMI VCDs (equation 1 in Material and Methods). As a result, there may be lags between the TROPOMI and the FLAME time series. This appears to be the case, for instance, for 7 July with a lag of ~30 minutes (Fig. 4).

**Figure 5:**
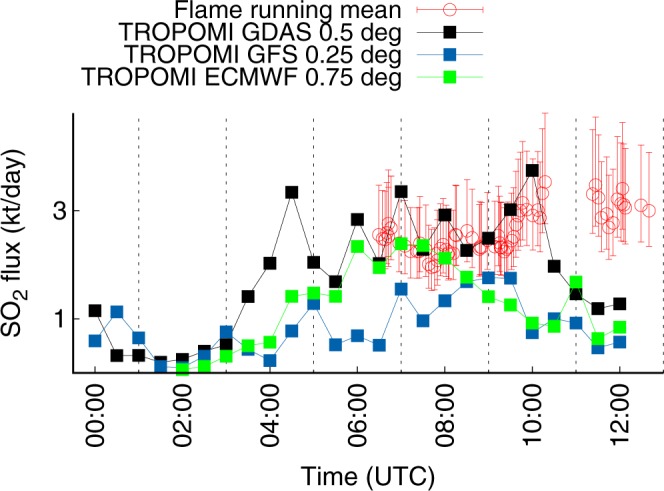
PlumeTraj results for different meteorological data sets compared with fluxes from FLAME. Example for 5 July 2018.

Since it concerns the precision rather than accuracy, even a relatively small error contribution from back-propagation analysis does not imply that its impact on the agreement is small. Errors in back-propagation mainly result from uncertainties in meteorological data, due to limited grid resolution, temporal resolution and the accuracy of the meteorological input data of PlumeTraj (assimilated empirical data versus modeled forecast data). Inaccurate meteorological data may lead to drastic decrease in accuracy of the flux time series with respect to the reference data (FLAME in this case). On the other hand, a high-resolution wind field data set increases computational costs significantly. We tested different meteorological data sets of different grid resolution (Fig. 5): Global Forecast System with quarter degree resolution (GFS 0.25°), Global Data Assimilation System with 0.5° resolution (GDAS 0.5°) and European Centre for Medium-Range Weather Forecasts with 0.75° resolution (ECMWF 0.75°). We found large variations as function of wind field data in the back-propagation result (accuracy) as shown in Fig. 5. For all days the best match with FLAME data was achieved with GDAS 0.5°, which consequently was used for the analysis. The poor match when using GFS 0.25° data is likely due to the forecast nature of the data while GDAS contains measured data.

As expected, the other major source of uncertainty impacting the TROPOMI derived fluxes were found to be clouds (Fig. 3c). The higher the cloud fraction associated with clouds overlying the SO_2_ plume the more this impedes the successful retrieval of SO_2_ concentrations. Clouds underlying the SO_2_ plume may actually enhance retrieval precision. In general, for cloud fraction above ~0.4 and when co-located with at least ~20% of the plume area (July 10, 23, 24, 26, August 1, 2, 4) an underestimated SO_2_ flux from TROPOMI was observed (Figs 3a,c and 4). For example, the interrupted SO_2_ cloud spreading towards West on the 23 July is interrupted by dense cloud cover (Fig. 2c), which in all likelihood caused the drop in SO_2_ flux to below 1 kt/day after 6h30 AM (Fig. 4). Dense cloud cover also appeared on 1 and 2 August, which possibly caused an underestimated SO_2_ flux with respect to the FLAME result (Figs 3 and 4). Notwithstanding the extensive cloud cover there appears to be a shift fairly constant in time, indicating coherence between the FLAME and TROPOMI time series (Fig. 4). Despite widely clear sky conditions and the fact the plume was fairly well recovered by PlumeTraj, the match is poor for the 29, 30 and 31 of July. Dense clouds at altitudes between ~2000 and ~3500 m occurred between 27 and 31 July, but only within a radius of ~15 km around the vents (Figs 1a,b and 3). Cloud cover over the vent affects all pixels and thus may lead to a significantly underestimated SO_2_ load, contributing to an underestimated flux.

### SO_2_ fluxes from TROPOMI versus driving traverse

Car-based traverses of the plume by UV spectrometer have been carried out during two days (Fig. 1c). The overpass of the Sentinel-5 swath around noon and the necessity of performing the traverse after sunrise limited the number of traverses per day to about 5 or 6. The weather conditions during measurements were typically cloudy, with periods of rain and fog. The cloud was low lying, with the summit of Etna and the plume mostly above the clouds (Fig. 1d,e).

**Figure 6:**
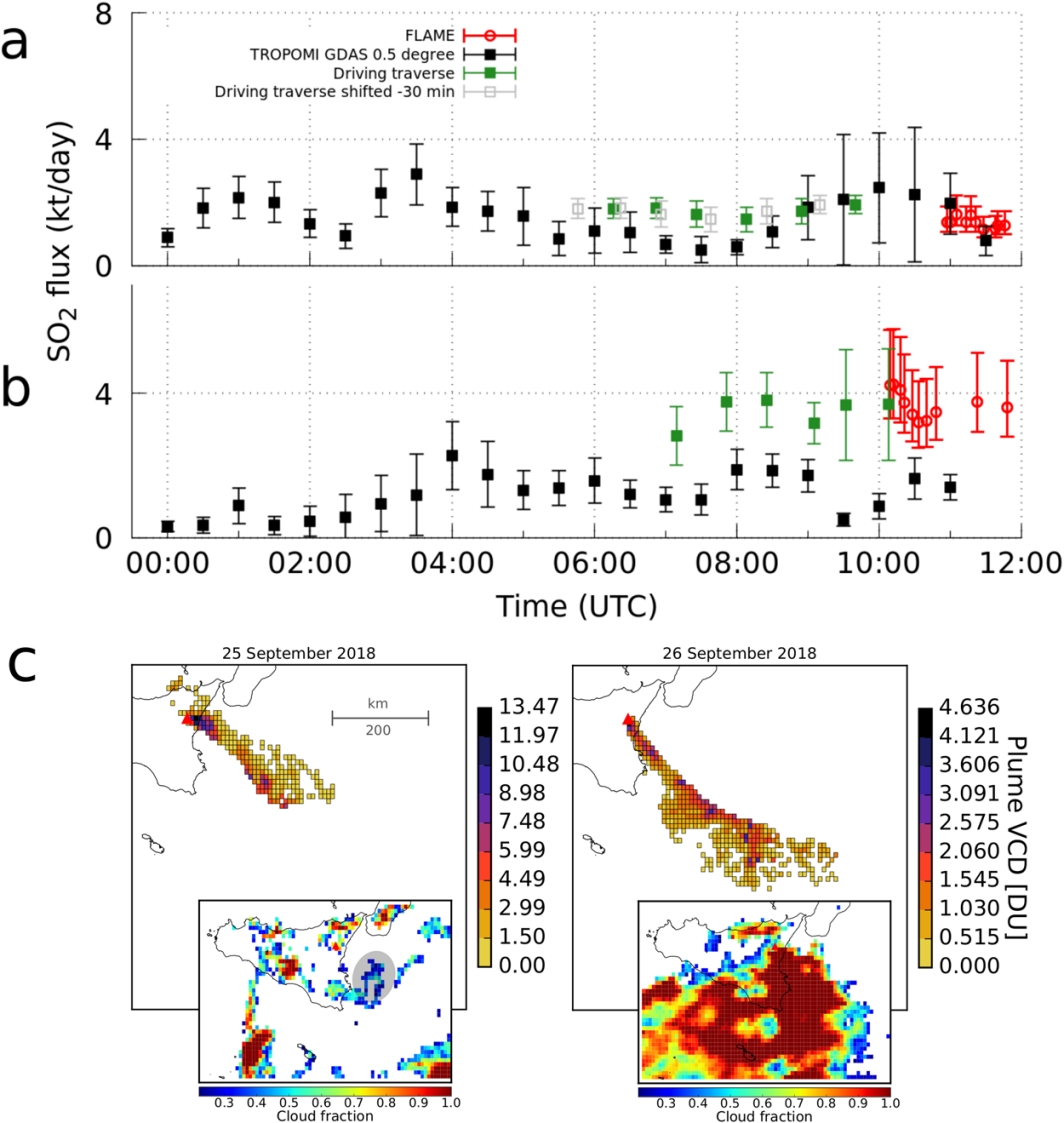
Comparison between TROPOMI derived SO_2_ fluxes with data from driving traverse measurements. (**a**) 25 September 2018. Also shown is the shifted time series from the traverse (grey boxes). (**b**) 26 September 2018. (**c**) Corrected VCDs from PlumeTraj and cloud fractions above 0.2. Grey shaded area marks cloudy period on 25 September between ~6 and 8h30 AM.

The results are shown in Fig. 6a,b. As with TROPOMI post-processing, the plume speed uncertainty retrieved from the optical flow analysis is prone to a fairly large error propagating into a fairly large flux uncertainty of ~25% (Material and Methods). For both days the time series are temporally coherent. The six traverses on 25 September yielded fluxes between 1.5 and 1.9 kt/day, in agreement with TROPOMI derived fluxes, except for the fluxes of the second and third traverse (between ~6h50 and 7h30 AM) which are substantially higher than the fluxes from TROPOMI data (around 0.6 kt/day, Fig. 6a). FLAME unfortunately yielded data only after 9h45 that day (Fig. 6a), which seem to follow the same trend as fluxes from TROPOMI data and the traverse measurement. Most of the meteorological cloud cover was lying below the plume, so SO_2_ overestimation from multiple scattering within the plume (light dilution) is unlikely for both FLAME and the driving traverse, but an underestimation is possible. This makes underestimated fluxes from TROPOMI even more likely.

Using the retrieved injection times from PlumeTraj to constrain the timing of cloud cover on 25 September results in significant cloud cover only occurring between ~6 and ~8h30 AM, with cloud fractions between 0.4 to 0.6 at around 2000 m altitude and clear sky after that (Fig. 6c), which is in line with the timing of the mismatch and could explain the underestimated TROPOMI result before ~8h30 AM, namely by masking the lower parts of the SO_2_ plume from TROPOMI (Fig. 6a). The time series from TROPOMI appears to be roughly 30 min ahead, a lag which is within the injection time uncertainty from PlumeTraj (Material and Methods). Shifting the traverse time series by −30 min would, however, still give an underestimated TROPOMI result for the cloudy period between ~6 and ~8h30 AM (Fig. 6a).

For 26 September, the ground-based time series is shifted to higher fluxes compared to the TROPOMI time series (Fig. 6b). Both follow a similar trend, which is comparable to the situation on days 1 to 3 August (Fig. 4), were similar overcast conditions dominated. Low lying (~1000 m to ~2500 m altitude) and dense cloud cover on 26 September (cloud fraction 0.6 to 1, Figs 6c and 1e), co-located with the plume, appears to have affected parts of the SO_2_ plume by attenuating light, which possibly has caused underestimated SO_2_ VCDs from TROPOMI.

In addition, during the traverse on the 26 September a splitting of the plume could be observed. Through analyzing the video footage used for the plume speed retrieval (Material and Methods) a significant wind shear between the plume and the higher meteorological clouds was found, which supports the observed plume splitting. This could have caused a dilution of SO_2_ below the pixel detection limit for some pixels and contributed to underestimated fluxes from TROPOMI when compared with ground-based measurements (Fig. 6b).

Cloud cover as a main factor causing the mismatch between the TROPOMI derived fluxes and those from the traverses is further suggested by the fact that fluxes from the driving traverse measurement agree with fluxes from FLAME, and fluxes from FLAME usually agree with fluxes from TROPOMI (Figs. 3 and 4).

## Discussion

TROPOMI and FLAME approaches have quite different measurement and processing steps to derive SO_2_ flux time series. Both TROPOMI and FLAME theoretically probe the full SO_2_ column in their respective field of view and both detect VCDs. However, their fields of view differ, so they probe different air masses with different sensitivities (not to mention differences in optical components and electronics). While the telescope of a FLAME scanner has a field of view of ~24 m at a typical distance to the plume of 3000 m, a TROPOMI pixel images an average concentration over an area of about 7 × 3.5 km^2^. FLAME measures SO_2_ columns near the source, whilst the TROPOMI data used here contains SO_2_ concentrations up to 12 h after emission, where VCDs and hence signal-to-noise is lower than near the crater and may dilute to concentrations below the detection limit of TROPOMI.

PlumeTraj does not currently account for chemical evolution of the plume. Airborne *in-situ* measurements in the Mt. Etna plume yielded insignificant conversion to sulfate aerosol in the first 5 h after injection^34^. During clear sky summer days SO_2_ to H_2_SO_4_ conversion may occur with volume concentration changes at rates of a few %/h^35^, which may make quite modest contributions to underestimated fluxes particularly for clear days and close to noon (e.g. 5, 6, 11, 16, 17, 19, 22 July, Fig. 4).

For cloudy days, cloud heights usually varied between ~1000 m, especially near the vent (Fig. 1e), and ~4500 m further downwind. This was the case especially between 27 and 31 July and as discussed for the driving traverse measurements. Clouds were thus vertically co-located with considerable parts of the plume so that scavenging of SO_2_ by cloud droplets^36^ would take place. A rough estimation assuming sulfur diffusion rates into droplets between 10^−8^ and 10^−4^ mol/l/s^37^, a vertical plume thickness of 100 m, mean droplet radius of 5 µm and density of 100/cm^3^ for stratocumulus clouds^38^ yields a negligible SO_2_ VCD loss rate below 10^−10^ DU/s.

Although ash emissions occurred, particularly after 23 August, these emissions had very localized impacts (~100 m) and did not rise above a few hundred meters from the crater. Uncertainties in TROPOMI VCDs due to ash emission can thus be neglected, including adsorption of SO_2_ on ash particle^39^.

Bias due to aerosol is not explicitly treated in the data processing^25^. Aerosol extinction is highly variable and a function of aerosol properties (e.g. size distribution). However, the cloud detection algorithm is expected to overestimate the cloud fraction in the presence of aerosols (if at similar heights), so that at least the non-absorbing part of aerosol extinction is accounted for.

While the spatiotemporal resolution of the meteorological data yields satisfactory agreement overall, much of the uncertainty of the back-trajectory analysis may indeed be attributed to the spatial and/or temporal wind field resolution being too low for some cases. An underestimation occurs when pixels which are clearly containing SO_2_ do not back-propagate to the vent, due to the limited temporal resolution of the wind field; this appears to be evident for 25 September (Fig. 6a). This particularly applies for more complicated plumes with small-scale vertical or horizontal wind shear, which includes the days 10 July and 2, 3, 4 August.

An overestimated (or underestimated) plume height by PlumeTraj may cause underestimated (or overestimated) fluxes. Figure 2a shows an example for 13 July. A considerable number of plume-pixels at the eastern flank of the plume are associated with the maximum modeled plume height of 7 km, which seems too high for quiescent degassing and thus could be erroneous, contributing to underestimated SO_2_ loads and thus fluxes.

An overestimation in flux may occur due to pixels that are visually not part of the plume, but are back-propagated to the vent (eastern plume flank, Fig. 2a; northern plume flank Fig. 2b). All three error sources (wrong trajectory, cloud cover, missing pixels) may contribute simultaneously, which, based on the PlumeTraj results and cloud fraction data, was likely the case for 27 July and 3 and 5 August.

Pixels near the swath edge have lower signal-to-noise since the TROPOMI detector binning is modified for these pixels to keep pixel sizes reasonable. This caused a relatively low signal-to-noise for 9, 20 and 25 July. The intra-diurnal trends are compatible for 20 and 25 July but the noise may have contributed to overestimated SO_2_ fluxes compared with the FLAME result.

Meteorological input data quality is an important issue also for other satellite remote sensing problems, in particular the spatial resolution of the wind field. Back-trajectory modeling was used to constrain CO_2_ enhancements detected by the Orbiting Carbon Observatory 2 (OCO-2) over Yasur volcano (Vanuatu)^40^ using wind field data with 0.5° grid resolution. The plume pixels were correctly back-propagated to the vent, even though the diameter of the Yasur vent is only ~200 m compared with ~1300 m at Mt. Etna.

The uncertainty of all three approaches (TROPOMI, FLAME, driving traverse) depends largely on how well the wind field can be constrained. The flux time series from FLAME is thus by no means an absolute reference for the TROPOMI result and prone to uncertainties that may be much larger than the −22% and +36% assumed here (Material and Methods). This is mainly due to an empirical relationship used between plume height and wind speed^29^. This fact motivated the driving traverse measurements, since VCDs can be retrieved without knowledge of the plume height. The result from the driving traverse is in line with the result from FLAME. This validates fluxes retrieved from the FLAME system, 12 years after its installation.

Clearly, a month of data is insufficient to study how the agreement between TROPOMI and FLAME varies over the course of a year and with the seasons, which is planned to be done once a year of data becomes available. To some extent, however, this has been assessed by the measurements in September where cloudy conditions dominated. Clouds are expected to have the largest impact on the flux retrieval from TROPOMI for the fall and winter months.

For validation purposes perhaps more significant than agreement of absolute fluxes at a given time is the substantial degree of temporal coherence between the flux time series derived from TROPOMI and FLAME, which is clearly the case, as it appears even for dense cloud cover (e.g. 1 August, 26 September).

In conclusion, after comparing SO_2_ fluxes from TROPOMI with fluxes from ground-based measurements we find that the agreement of the monthly average is excellent, the agreement between the daily averages is fairly good and the intra-diurnal agreement on a 30-min timescale is satisfactory. This indicates that using TROPOMI satellite images of SO_2_ columns and back-trajectory analysis with a fairly coarse wind field can deliver reliable fluxes of volcanic SO_2_ (and potentially other gases such as CO) in nearly real-time with high spatiotemporal resolution. This allows studying quiescent degassing processes of a tremendous number of volcanoes globally, providing new insights into the inner working of volcanoes, their risks, and their impact on atmospheric chemistry and physics. This leads to the main conclusion that the future allows for global automated real-time measurements of large numbers of degassing volcanoes world-wide, revolutionizing the quantity and quality of magmatic degassing data available to the volcanological community.

## Material and Methods

### Flux retrieval from TROPOMI satellite data using PlumeTraj

The spectral radiances acquired by passive satellite remote sensing instruments are strongly dependent on plume height, but lack altitude information. Thus, the L2 data products used for this study provide images containing SO_2_ vertical column densities (VCD) and assume a box of fixed height (altitude level) within which the SO_2_ is located. In total, there are three different altitude levels (lower troposphere: surface to 1 km, mid-troposphere: 6.5 to 7.5 km (Fig. 1b) and upper troposphere/lower stratosphere: 14.5 to 15.5 km), corresponding to three images. To retrieve accurate volcanic plume SO_2_ masses from these images the actual volcanic plume height and vertical extension have to be retrieved first. To that end a pixel-by-pixel numerical procedure called PlumeTraj is used, which is fully detailed in the references^32,33^.

PlumeTraj integrates the Hybrid Single-Particle Lagrangian Integrated Trajectory model (HYSPLIT^41^) using custom-built routines written in the Python programming language. The scheme selects those pixels, which are associated with trajectories emerging from the location of the volcanic vent (plume pixels). For each of these pixels PlumeTraj computes the height (above sea level, asl) at which the SO_2_ is located at satellite measurement time instant (plume height), the height (asl) at which the prevailing atmospheric winds starts to disperse the gas into the atmosphere (injection height) and the time when the SO_2_ reaches the injection height (injection time, Fig. 2). The retrieved plume height is used to correct the VCDs of each pixel of the satellite images. From the three quantities and the corrected VCDs the SO_2_ load is computed for each plume pixel. To compute a flux time series used in the comparison, time bins of 30 minutes length are defined. Pixels with injection times matching a given bin time are combined. The integrated SO_2_ load (sum of SO_2_ mass of all pixels per bin) divided by the bin length yields the flux for a given time bin. The uncertainty of the SO_2_ flux then results from the error in SO_2_ mass and injection time. Assuming they are uncorrelated, the relative error of the flux for a given time instant (bin) is computed as

1 $$\delta {F}^{2}=\delta {m}^{2}+\delta {\rm{\Delta }}{t}^{2},$$

where *F* is the SO_2_ flux computed by PlumeTraj, *m* is the integrated SO_2_ mass and ${\rm{\Delta }}t={t}_{i+1}-{t}_{i}$ the length of a time bin between two subsequent injection times and *t*_*i*_ and *t*_*i*+1_ with an associated relative uncertainty calculated as

2 $$\delta {\rm{\Delta }}t=\frac{{\sigma }_{{t}_{i+1}}-{\sigma }_{{t}_{i}}}{{\rm{\Delta }}{t}_{i}},$$

where ${\sigma }_{{t}_{i+1}}\,\,$and ${\sigma }_{{t}_{i}}$ are the mean standard deviations of the injection times (in minutes) of two subsequent time instants and quantify the uncertainty of the start and end time of a given bin *i*. The relative uncertainty of the SO_2_ mass *δm* is directly proportional to the error in VCD and evaluated as

3 $$\delta {m}^{2}=(\frac{{{\sigma }_{h}}^{2}+{{\sigma }_{r}}^{2}}{n}+{{\sigma }_{s}}^{2})\frac{1}{VC{{D}_{c}}^{2}}\,$$

where *n* is the number of pixels per bin, *VCD*_*c*_ is the corrected VCD (mean over *n*) using the plume heights *h* and *σ*_*h*_ the random error (mean over *n*) of the plume height from PlumeTraj alone, which typically varies between 0 m and 2 km for a given plume. *σ*_*h*_ quantifies the uncertainty of VCD introduced by PlumeTraj during the correction of VCDs due to uncertainty in the plume height. *σ*_*r*_ is the random error and *σ*_*s*_ the systematic error (both mean over *n*) of TROPOMI data contained in the L2 data product file, accounting for twenty sources of error^25^. For instance, *σ*_*r*_ includes shot noise, and uncertainty due to cloud fraction, *σ*_*s*_ includes the error introduced by the radiative transfer forward model and uncertainties of spectroscopic parameters such as SO_2_ absorption cross section and a contribution from plume height uncertainty, which may lead to a slight overestimation of the total uncertainty in equation 1. *σ*_*h*_ and ${\sigma }_{{t}_{i}}$ are estimated from a sensitivity analysis of the distance of approach of the trajectories^32^ and are a measure of the accuracy of the back-trajectory analysis performed by PlumeTraj.

TROPOMI data were regridded onto a 0.07° × 0.07° grid (Fig. 2). For a given satellite image (one per day), the back-trajectory modeling was carried out between 1500 and 7000 m asl and from the time of acquisition (around noon UTC) up to 12 h backwards in time. After that, SO_2_ is usually largely diluted below TROPOMI detection limit. To speed up the analysis, noisy pixels were excluded from the analysis by applying a threshold VCD of 0.4 DU to the input satellite data (Fig. 2).

Apart from satellite data PlumeTraj needs a meteorological dataset (including the wind field) as input. Here, data from the Global Data Assimilation System (GDAS) with 0.5° resolution were used.

### Ground-based SO_2_ flux measurements with the FLAME network

The FLAME network consists of nine ultraviolet scanning spectrometers spaced ~7 km apart and installed at a mean altitude of ~900 m asl on the flanks of Mt Etna (Fig. 1c). Each station scans the sky over 156°, intersecting the plume at a mean distance of ~14 km from the summit craters^29^. The network produces measurements on 86% of days per year. At each scanner, diffuse sky radiation is received through a filter for visible light (HOYA U330) and then reflected by a 45° plane mirror into a telescope of 8 mrad instrumental field-of-view (FOV). The beams are then focused into fiber optics cables connected to S2000 Ocean Optics spectrometers with UV output in the 295 to 375 nm spectral range. The system is collecting data daily for almost 9 h depending on the season and acquiring a complete scan (105 spectra including a dark spectrum) in ~5 min. Open-path ultraviolet spectra are converted to SO_2_ column amounts on site applying the DOAS technique and using a modeled clear-sky spectrum^29^. Inverted data are transmitted to INGV Catania where SO_2_ emission rates are computed by multiplying by the wind speed^29^.

There is a trade-off between number of scanners and accuracy of plume height, which is needed to compute SO_2_ column densities as accurately as possible. Errors due to plume-height uncertainties arising from sparse scanner density is somewhat masked by both the large natural variability in the SO_2_ flux emitted from Etna and the inherent error in wind speed. Uncertainties in SO_2_ flux by stationary automatic scanning array arise from several sources, such as: (i) DOAS retrieval using a modelled reference spectrum (12%)^29^; (ii) multiple scattering effects (±10%); (iii) plume speed (±10 up to ±20%); (iv) height of the plume (±10 to ±20%) due mainly to wind speed error; (v) and evaluation of the flux using the slant column amounts (15%)^42^. Errors in geometric corrections are negligible. The sum of the individual errors, as square root of the sum of the squares of the individual errors, yields a total uncertainty between −22 and +36%, which is adopted here.

Since PlumeTraj calculates fluxes at the vent location, all FLAME-based flux time series have been shifted in time by –*d*/*v* where *d* is the mean distance between vent and flame network and *v* is the wind speed.

### Ground driving traverse

Ground-based traverse measurements were performed on two days along roads as close to the volcano as possible (Fig. 1c). These follow a similar method to the stationary scanners, but instead of measuring the plume cross-sections by scanning across the sky, a zenith pointing spectrometer is moved across the plume, preferably close to orthogonally to the plume direction. For these measurements a collimating telescope (mounted to a car) collected the diffuse sky light and focused it into a fiber optic cable which was connected to an Ocean Optics USB2000 + spectrometer inside the car. These spectra were analyzed in real-time using the iFit method^43^ to retrieve the SO_2_ vertical column amounts (VCA). The position of each spectrum is recorded using GPS. The VCA from each spectrum is multiplied by the distance travelled, corrected for a non-orthogonal direction of travel with respect to the plume direction, which is defined as the vector from the crater to the center-of-mass of the plume. These are then summed over the traverse to give the SO_2_ plume cross-section, which is converted to a flux by multiplying by the wind speed, as with the FLAME network. The spectra were acquired at a frequency of ~0.2 Hz, varying the integration time and number of spectra that are averaged to keep the temporal resolution constant while maximizing the signal-to-noise in the spectrum. Traverses were performed with a 30–45 minutes period, both in a clockwise and anti-clockwise direction around Etna to avoid errors from changes in the plume azimuth during the traverses.

Although the temporal resolution of the traverses is much lower than the FLAME network, the spectra are measured pointing at zenith so some of the sources of error are removed (specifically from the plume height and use of slant columns). Errors from the retrieval scheme, multiple scattering and the wind speed still remain. To help constrain the wind speed (and uncertainty), images from the INGV camera permanent network were used to measure the plume speed during the traverses. The camera was located at Montagnola (Fig. 1c,d, latitude: 37.719° N, longitude: 15.0036°E, ~2600 m asl), above the level of the cloud. The footage is first masked to select the plume, after which the Farnebäck optical flow algorithm is applied to produce flow maps of the plume. Corrections are applied to take the plume azimuth and pixel size into account. The uncertainty in the wind speed was typically 20–25%, depending on the absolute speed and uncertainty (typically errors are much larger for slower wind speeds). This gives a total uncertainty of approximately ±25%. As with FLAME, the time series have been shifted in time to correct for the distance between vent location and flame network.

## Data Availability

The TROPOMI data version used for the analysis and the FLAME data is available upon request from the corresponding author or N. T. The current description of the L2 product retrieval algorithm for SO_2_ is available at: http://www.tropomi.eu/sites/default/files/files/S5P-BIRA-L2-ATBD-SO2_400E_v1.1.0_20181005.pdf.

## References

[1] Scaillet B, Clemente B, Evans BW, Pichavant M (1998). Redox control of sulfur degassing in silicic magmas. J. Geophys. Res. Solid Earth.

[2] Shinohara H (2008). Excess degassing from volcanoes and its role on eruptive and intrusive activity. Rev. Geophys..

[3] Allard P (1997). Endogenous magma degassing and storage at Mount Etna. Geophys. Res. Lett..

[4] Sutton AJ, Elias T, Gerlach TM, Stokes JB (2001). Implications eruptive processes as indicated by sulfur dioxide emission from Kîlauea Volcano, Hawai’i, 1979–1997. J. Volcanol. Geotherm. Res..

[5] Edmonds, M., Oppenheimer, C., Pyle, D., Herd, R. & Thompson, G. SO_2_ emissions from Soufrière Hills volcano and their relationship to conduit permeability, hydrothermal interaction and degassing regime. *J. Volcanol. Geotherm. Res.* **124**, 23–43 (2003a).

[6] Palma JL (2008). Correlations between SO_2_ flux, seismicity, and outgassing activity at the open vent of Villarrica volcano, Chile. J. Geophys. Res. Solid Earth.

[7] Caltabiano, T. *et al*. Volcanic gas emissions from the summit craters and flanks of Mt. Etna, 1987–2000,” In *Mt. Etna: Volcano Laboratory*, **143**, Bonaccorso, A., Calvari, S., Coltelli. M., Del Negro, C. & Falsaperla, S., Washington, DC: American Geophysical Union, 111–128, 10.1029/143GM08 (2004).

[8] Burton MR, Caltabiano T, Murè F, Salerno G, Randazzo D (2009). SO_2_ flux from Stromboli during the 2007 eruption: Results from the FLAME network and traverse measurements. J. Volcanol. Geotherm. Res..

[9] Burton MR (2013). Sawyer & G. M., Granieri, D. Deep Carbon Emissions from Volcanoes. Rev. Min. Geochem..

[10] Stoiber R. E, Malinconico J. L. L. & Williams, S. N. Use of the correlation spectrometer at volcanoes. In: Tazieff, H., Sabroux, J.C. (eds), Forecasting volcanic events. Elsevier, New York, 424–444 (1983).

[11] Horton K (2006). Real-time measurement of volcanic SO_2_ emissions: Validation of a new UV correlation spectrometer (FLYSPEC). Bull. Volcanol..

[12] Mori T, Burton M (2006). The SO_2_ camera: A simple, fast and cheap method for ground‐based imaging of SO_2_ in volcanic plumes. Geophys. Res. Lett..

[13] Rodríguez LA (2004). SO_2_ emissions to the atmosphere from active volcanoes in Guatemala and El Salvador, 1999- 2002. J. Volcanol. Geotherm. Res..

[14] Galle, B. *et al*. Network for Observation of Volcanic and Atmospheric Change (NOVAC)-A global network for volcanic gas monitoring: Network layout and instrument description. *J. Geophys. Res*. **115**, D05304, 10.1029/2009JD011823.

[15] NAS (National Academies of Sciences). Volcanic Eruptions and Their Repose, Unrest, Precursors, and Timing. Washington, DC: The National Academy Press. doi: 10.17226/24650, https://www.nap.edu/catalog/24650/volcanic-eruptions-and-their-repose-unrest-precursors-and-timing (accessed 10 May 2018) (2017).

[16] Laiolo M (2018). Long-term eruptive trends from space-based thermal and SO_2_ emissions: a comparative analysis of Stromboli, Batu Tara and Tinakula volcanoes. Bull. Volcanol.

[17] Taran Y (2018). Gas Emissions From Volcanoes of the KurilIsland Arc (NW Pacific): Geochemistry and Fluxes. Geochem. Geophys. Geosys..

[18] Prata AJ (2009). Satellite detection of hazardous volcanic clouds and the risk to global air traffic. Nat. Hazards.

[19] Carn SA, Fioletov VE, McLinden CA, Li C, Krotkov NA (2017). A decade of global volcanic SO_2_ emissions measured from space. Sci. Rep..

[20] Furtney MA (2018). Synthesizing multi-sensor, multi-satellite, multi-decadal datasets for global volcano monitoring. J. Volcanol. Geotherm Res..

[21] Carn, S.A. et al. Volcanic eruption detection by the Total Ozone Mapping Spectrometer (TOMS) instruments: a 22-year record of sulfur dioxide and ash emissions. In: Oppenheimer, C., Pyle, D. M. & Barclay, J. (eds), Volcanic Degassing, Geological Society, London, *Special Publications*2**13**, 177–202 (2003).

[22] Hassinen S (2016). Overview of the O3M SAF GOME-2 operational atmospheric composition and UV radiation data products and data availability. Atmos. Meas. Tech..

[23] Clarisse L (2012). Retrieval of sulphur dioxide from the infrared atmospheric sounding interferometer (IASI). Atmos. Meas. Tech..

[24] Carn, S. A., Krotkov, N. A., Yang, K. & Krueger, A. J. Measuring global volcanic degassing with the Ozone Monitoring Instrument (OMI), Geological Society, London, *Special Publication*s **380**, 11 July 2013, 10.1144/SP380.12 (2013).

[25] Theys N (2017). Sulfur dioxide retrievals from TROPOMI onboard Sentinel-5 Precursor: algorithm theoretical basis. Atmos. Meas. Tech..

[26] Theys, N. *et al*. Global monitoring of volcanic SO_2_ degassing from space with unprecedented resolution. *Sci. Rep*., submitted (2018). 10.1038/s41598-019-39279-y PMC6390096 30804392

[27] Theys N (2013). Volcanic SO_2_ fluxes derived from satellite data: a survey using OMI, GOME-2, IASI and MODIS. Atmos. Chem. Phys..

[28] Bauduin S, Clarisse L, Clerbaux C, Hurtmans D, Coheur P-F (2014). IASI observations of sulfur dioxide (SO_2_) in the boundary layer of Norilsk. J. Geophys. Res. Atmos..

[29] Salerno GG (2009). Three-years of SO_2_ flux measurements of Mt. Etna using an automated UV scanner array: Comparison with conventional traverses and uncertainties in flux retrieval. J. Volcanol. Geotherm Res..

[30] Pugnaghi S, Gangale G, Corradini S, Buongiorno MF (2006). Mt. Etna sulfur dioxide flux monitoring using ASTER-TIR data and atmospheric observations. J. Volcanol. Geotherm Res..

[31] Carboni E (2016). The vertical distribution of volcanic SO_2_ plumes measured by IASI. Atmos. Chem. Phys..

[32] Pardini F (2017). Retrieval and intercomparison of volcanic SO_2_ injection height and eruption time from satellite maps and ground-based observations. J. Volcanol. Geotherm Res..

[33] Pardini F, Burton M, Arzilli F, La Spina G, Polacci M (2018). SO_2_ emissions, plume heights and magmatic processes inferred from satellite data: The 2015 Calbuco eruptions. J. Volcanol. Geotherm Res..

[34] Voigt C (2014). Evolution of CO_2_, SO_2_, HCl, and HNO_3_ in the volcanic plumes from Etna. Geophys. Res. Lett..

[35] Cheng L, Peake E, Davis A (1987). The Rate of SO_2_ to Sulfate Particle Formation in an Air Parcel from an Oil Sands Extraction Plant Plume. JAPCA.

[36] Textor C, Graf H-F, Herzog M, Oberhuber JM (2003). Injection of gases into the stratosphere by explosive volcanic eruptions. J. Geophys. Res..

[37] Waltrop A, Mitra SK, Flossmann AI (1991). & Pruppacher, On the Scavenging of SO_2_ by Cloud and Rain Drops: IV A Wind Tunnel and Theoretical Study of the Absorption of SO_2_ in the ppb(v) Range by Water Drops in the Presence of H_2_O_2_. J. Atmos. Chem..

[38] Woods, R. Stratocumulus Clouds. *Mon. Wea. Rev*., **140**, 2373–2423, 10.1175/MWR-D-11-00121.1 (2012).

[39] Schmauss D, Keppler H (2014). Adsorption of sulfur dioxide on volcanic ashes. American Mineralogist..

[40] Schwandner F (2017). Spaceborne detection of localized carbon dioxide sources. Science.

[41] Stein AF (2015). NOAA’s HYSPLIT Atmospheric Transport and Dispersion Modeling System. BAMS.

[42] Edmonds M, Herd RA, Galle B, Oppenheimer C (2003). Automated, high time-resolution measurements of SO_2_ flux at Soufrière Hills Volcano, Montserrat. Bull. Volcanol..

[43] Esse, B., Burton, M., Varnam, M., Kazahaya, R. & Salerno, G. iFit: An intensity based retrieval for volcanic SO_2_ from scattered sunlight UV spectra. *Atmos. Meas. Tech. Discuss.*, 10.5194/amt-2018-404, in review, 2018.

